# Translation, cultural adaptation and psychometric testing of Igbo fear avoidance beliefs questionnaire in mixed rural and urban Nigerian populations with chronic low back pain

**DOI:** 10.1371/journal.pone.0216482

**Published:** 2019-05-14

**Authors:** Chinonso Nwamaka Igwesi-Chidobe, Charity Amarachukwu, Isaac Olubunmi Sorinola, Emma Louise Godfrey

**Affiliations:** 1 Department of Medical Rehabilitation, Faculty of Health Sciences and Technology, College of Medicine, University of Nigeria (Enugu Campus), Nigeria; 2 Department of Physiotherapy, School of Population Health Sciences, Faculty of Life Sciences and Medicine, King’s College London, London, United Kingdom; 3 Department of Physiotherapy, University of Nigeria Teaching Hospital, Enugu, Nigeria; 4 Department of Psychology, Institute of Psychiatry, Psychology and Neuroscience, King′s College London, United Kingdom; Public Library of Science, UNITED KINGDOM

## Abstract

**Introduction:**

Low back pain (LBP) is highly prevalent in Nigeria and is more devastating in rural Nigeria due to adverse living and working conditions, reinforced by maladaptive illness beliefs. There is a need to develop measures for assessing such beliefs in this population. This study aimed to cross-culturally adapt the Fear Avoidance Beliefs Questionnaire (FABQ) and test its psychometric properties in mixed rural and urban Nigerian populations with chronic LBP.

**Methods:**

Translation, cultural adaptation, test–retest, and cross-sectional psychometric testing. FABQ was forward and back translated by clinical/non-clinical translators. A review committee evaluated the translations. Twelve people with chronic LBP in a rural Nigerian community pre-tested the questionnaire. Cronbach’s alpha assessing internal consistency; intra-class correlation coefficient and Bland–Altman plots assessing test–retest reliability; and minimal detectable change were investigated in a convenient sample of 50 chronic low back pain sufferers in rural and urban Nigeria. Construct validity was examined using Pearson’s correlation analyses with the eleven-point box scale and Igbo Roland Morris Disability Questionnaire (Igbo-RMDQ), and exploratory factor analysis in a random sample of 200 adults with chronic low back pain in rural Nigeria. Ceiling and floor effects were investigated in all samples.

**Results:**

Amendments allowed interviewer-administration. Item 8 was modified to ‘I have a compensation or gains I get from having my pain’ as there is no benefit system in Nigeria. Igbo phrase for ‘physical activity’ could also mean ‘being active’, ‘moving the body’ or ‘moving about’ and was used in the items with ‘physical activity’. The Igbo-FABQ had good internal consistency (α = 0.80–0.86); intra class correlation coefficients (ICC = 0.71–0.72); standard error of measurements (3.21–7.40) and minimal detectable change (8.90–20.51). It correlated moderately with pain intensity and disability, with a two-factor structure and no floor and ceiling effects.

**Conclusions:**

Igbo-FABQ is valid, reliable, and can be used clinically and for research.

## Introduction

Global burden of disease studies indicate that low back pain (LBP) is the major cause of years lived with disability in developed and developing countries [1–4]. The one-year prevalence rate of 40–85% in Nigeria is greater than 14–51% reported in other African countries [5–7]. The point prevalence rate of 33–40% in Nigeria is greater than the 10–33% in western developed countries including the United Kingdom, Canada and Belgium [3, 8]. The burden of LBP is unduly greater in rural Nigeria with one-year prevalence rates 70–85% [6, 7, 9, 10]. In contrast, the one-year prevalence rates of LBP range between 40–47% in urban Nigeria [6, 11].

The fear avoidance model posits that some individuals avoid activities believed to cause pain, even when they are neither harmful nor painful, which leads to disuse, deconditioning and poor performance of physical tasks [12–17]. Fear avoidance beliefs have been associated with LBP in high income countries, and are consistent predictors of chronicity, LBP disability and failure to return to work, in systematic reviews [18–20], and state of the art reviews [12–14, 16, 21]. A systematic review with clearly defined work and non-work disability outcomes has also shown that fear avoidance beliefs are mediators and moderators of treatment efficacy (return to work, perceived disability and pain) in patients with back pain [20].

Only a few studies have studied the influence of fear avoidance beliefs in Africa. Work-related fear avoidance beliefs were associated with LBP disability in 366 South African steel plant workers involved in manual labour [22]. However, the involvement of mostly males in an urban African occupational setting limits generalisability to other African populations. In rural African contexts, studies have not investigated the influence of fear avoidance beliefs on LBP disability. In Nigeria, most research has involved urban English speaking participants, precluding the illiterate rural dwellers with the worst health outcomes [11, 23–28]. This exclusion could be due to the possible need to adapt English self-report measures into native interviewer-administered measures for illiterate rural dwellers which may be more tasking and complicated [29]. However, evidence suggests that validity of interviewer-administration of self-report measures is ensured when interviewers are adequately trained to eliminate or significantly reduce bias to patient responses [30, 31]. Furthermore, interviewer-administration reduces the likelihood of missing data [31], and may be the only available method for administering self-report measures to people with low levels of literacy in resource constrained places [32–34].

A qualitative study in rural Nigeria showed that people viewed LBP as a ‘disease of hard labour’ suggesting that fear avoidance beliefs may be important in this context [35]. A subsequent preliminary cross-sectional survey suggested that fear avoidance beliefs were associated with disability in rural Nigeria [36]. However, similar to other rural African contexts, there are no measures to assess fear avoidance beliefs in rural Nigeria. This study aimed to cross-culturally adapt and validate the Igbo version of the FABQ in mixed rural and urban Nigerian populations.

## Methods

### Ethical approval and consent to participate

Ethical approvals were obtained from King’s College London (Ref: BDM/13/14-99) and University of Nigeria Teaching Hospital (Ref: UNTH/CSA/329/Vol.5). Written informed consent was received from all participants prior to involvement in the study.

### Study designs

Translation, cultural adaptation, test-retest measurements and cross-sectional study of psychometric properties.

### Outcome measures

#### Fear avoidance beliefs questionnaire (FABQ)

The fear avoidance beliefs questionnaire (FABQ) is one of the best measures for assessing fear avoidance beliefs [17]. It is a sixteen-item back pain-specific self-report measure that assesses the extent to which pain is believed to be caused or aggravated by general physical activity (FABQ-PA) and work-related activities (FABQ-W). These represent the two subscales of the measure [17]. Summing the two subscale scores gives a total FABQ score of 66, with higher scores reflecting stronger fear avoidance beliefs [17]. FABQ-PA has five items, each scored with a Likert scale ranging from 0 (completely disagree) to 6 (completely agree). For the original English FABQ, participants were instructed to circle any number from 0 to 6. One item (1) is a distractor and is not scored. The maximum score for FABQ-PA is 24 and the minimum is 0, with higher scores indicating stronger fear avoidance beliefs related to physical activity. FABQ-W has 11 items, each having a Likert scale ranging from 0 (completely disagree) to 6 (completely agree), but four items (8, 13, 14, 16) are distractors, and do not contribute to total score. The maximum score for FABQ-W is 42 and minimum score is 0 with higher scores indicating stronger fear avoidance beliefs related to work activities. FABQ correlates significantly with other measures of fear-avoidance such as the Tampa Scale of Kinesiophobia; r = 0.33–0.59 [37]. The internal consistency of FABQ range between 0.77 and 0.88 [17]. A change of 13 from baseline is reported to be clinically important [38].

#### Eleven-point box scale (BS-11)

BS-11 is a single eleven-point numeric scale for pain intensity [39, 40]. It is made up of eleven numbers (0 through 10) within boxes [41]. Zero means ‘no pain’ and 10 denotes ‘pain as bad as you can imagine’ or ‘worst pain imaginable’ [39, 42]. It was chosen due to its easy comprehensibility and simple administration [39], in this population where the simple VAS was not easily understood [35].

#### Igbo Roland Morris disability questionnaire

RMDQ was chosen because it is valid and is the most widely used measure of LBP disability [43]. It is the main outcome tool for standardising outcome assessment in LBP randomised controlled trials, meta-analyses, cost-effectiveness analyses and multi-site studies [44]. RMDQ is easily administered, easy to understand, and is the best used in primary care or population-based studies [44, 45].

The Igbo-RMDQ [46] was adapted from the original English RMDQ, a twenty-four item back specific self-report measure. Each item is scored either a 0 or 1 [47]. A total score of 24 is the maximum and signifies the highest possible disability level and 0 means absence of disability. The face and content validity, construct validity, internal consistency, test-retest reliability and responsiveness have been shown to be very good [45]. It’s Cronbach’s alpha ranges between 0.84 and 0.93. The test-retest reliability ranges between 0.72 and 0.91. A 2-3-point change from baseline has been shown to be clinically important [45]. The measure conceptualises disability at the three levels of the ICF: body structures and function, activities and participation, and environmental factors. Similar to other LBP-specific disability measures, it places less emphasis on participation, and does not capture work-related outcomes [48].

### Cross-cultural adaptation

The procedure used throughout this section have been used in the cross-cultural adaptation of other Igbo self-report measures. Therefore, the text reproduces some information that have been published elsewhere (46).

#### Participants

Participants were clinical translators, non-clinical translators, an expert review committee, and people living with chronic LBP (LBP lasting for over 3 months). The clinical translator was a physiotherapist with 12 years of experience practising clinically in Nigeria. The three non-clinical translators included two Igbo linguistic experts who were professional translators with experience in patient self-reported outcomes. A health psychologist and an academic physiotherapist practicing in the United Kingdom, and an Igbo clinical psychologist and an Igbo clinical physiotherapist practising in eastern Nigeria made up the expert review committee members.

Cognitive debriefing (also known as verbal pre-testing) of Igbo-FABQ was done with a convenience sample of adults with chronic LBP in a rural Nigerian population whose pain were not due to infection, inflammation, spinal fracture, cauda equina syndrome or malignancy [35]. They were informed about the study and informed consent was subsequently obtained.

#### Procedure for cross-cultural adaptation

**Fig 1:**
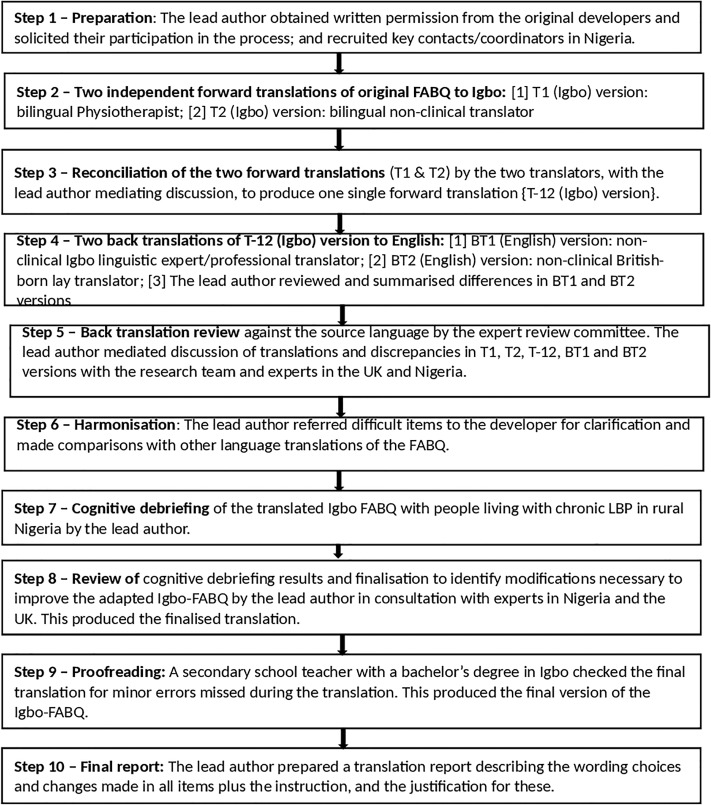
Cross-cultural adaptation stages.

The original FABQ [17] was cross-culturally adapted following generally accepted evidence-based guidelines [49, 50] to produce the Igbo-FABQ (Fig 1).

First stage–In August 2014, the lead author sought and obtained permission from Professor Gordon Waddell (now of blessed memory). He emphasized the need to collaborate with a team of experts including psychologists considering the technical and complex nature of translating and re-standardising a psychometric questionnaire into another language. The lead author adhered to his recommendations for translating the measure and also recruited the key contact persons and experts in Nigeria, the translators and the people living with chronic LBP.

Second stage–the questionnaire was forward translated from English to Igbo by one bilingual clinical physiotherapist and one bilingual non-clinical professional translator working independently. They were both native Igbo speakers fluent in the English language. Items were defined to enable the clinical translator to understand the assessed construct in order to provide psychometric equivalence with the original RMDQ. Items were not defined for the nonclinical translator to ensure that the translation reflected the lay language used in Igbo culture. This produced two Igbo FABQ versions: T1 and T2 respectively.

Third stage–T1 and T2 were reconciled via discussion between the two forward translators, mediated by the bilingual (English and Igbo) first author. This produced one Igbo FABQ version: T-12. Translations were compared and inconsistencies were recorded.

Fourth stage–the Igbo (T-12) version of the FABQ was back translated from Igbo to English by two back translators, unaware of the original version, who were from non-clinical occupations. One of them was an Igbo linguistic expert who translated tools professionally, and the other was a native English speaker, born in England but with Nigerian-born parents. This produced two back-translated English versions: BT1 and BT2. This step was a validation process which guaranteed a consistent translation that ensured the translated FABQ version (T-12) was reflecting the meaning in the original FABQ.

Fifth stage–T1, T2, T-12, BT1 and BT2 versions of the questionnaire were discussed by the expert review committee mediated by the lead author to produce an updated Igbo-FABQ version. This committee aimed to achieve cross-cultural equivalence in terms of semantic, idiomatic, experiential and conceptual equivalence [50]. Semantic equivalence was ensured by exploring Igbo and English words to assess if they meant similar things, if an item had multiple meanings, and if there were difficulties in the grammatical expressions used in the translations. Alternative Igbo idioms and colloquialisms were formulated where the English versions were difficult to translate to guarantee idiomatic equivalence. The expert review committee ensured that the FABQ items were experienced similarly in English and Igbo cultures to realise experiential equivalence. The words in the items, instructions, and response options were determined to have similar conceptual meanings in Igbo and English cultures which confirmed conceptual equivalence. The expert review committee determined that the Igbo wordings used were simple and could be easily understood in spite of age and educational levels.

Sixth stage–This was ‘harmonisation’ which involved reference to the cross-cultural adaptation reports of the Norwegian, Brazilian-Portuguese and German versions of the FABQ and critical appraisal of the fear avoidance model [12–14, 16] for any discrepancies with the translation and adaptation.

Seventh stage–involved cognitive debriefing of the translated Igbo FABQ by verbal pretesting among twelve participants living with chronic LBP in rural Nigeria [35]. The Igbo-FABQ was interviewer-administered by the lead author the using the ‘think-aloud’ cognitive interviewing style to assess comprehensibility, acceptability of items and cultural equivalence. The lead author read out each item and encouraged the participants to actively verbalise their thoughts as they tried to answer each question. The lead author asked participants if they encountered difficulty understanding the questionnaire, what they understood by each item, their perceived meaning of the chosen response, and if any item was found to be offensive by them. The lead author encouraged participants to keep talking while she recorded their responses. This seventh stage ensured that equivalence was maintained in the target setting–Nigeria to produce the final Igbo-FABQ, and this stage confirmed face and content validity [50].

Eight stage–involved a review of the cognitive debriefing results during which the lead author identified problematic items, statements, phrases and words in terms of comprehensibility, acceptability, and cultural equivalence. In consultation with Igbo and English linguistic experts, and Igbo and English physiotherapists and health psychologists, the lead author replaced problematic items, statements, phrases and words with more acceptable options.

Ninth stage–a secondary school Igbo teacher in a Nigerian school, cross-checked the Igbo-FABQ translation to eliminate any existing minor errors that may have been missed during translation and cultural adaptation. This produced the final Igbo-FABQ.

Final (tenth) stage–the lead author described the translation process, changes made to different sections of the original questionnaire, and justification of changes made, which are reported in this paper.

### Psychometric testing

The procedure used throughout this section have been used in the validity and reliability testing of other Igbo self-report measures. Therefore, the text reproduces some information that have been published elsewhere (46).

#### Participants

**Sample size estimation for test-retest reliability:** A study was carried out for test-retest reliability assessment. A minimum sample size of 27 was required to detect an intra-class correlation coefficient of 0.9 and a maximum width of 0.23 for the 95% confidence interval. This sample size calculation was informed by a previous reliability study in South Africa [51]. A convenience sample of 50 participants with chronic LBP, between the ages of 18 and 69 years, were recruited from communities in rural and urban areas of Enugu State, in the south-eastern part of Nigeria. They were informed about the study, screened and informed consent was then obtained.

**Sample size estimation for construct validity:** A sample size of 194 would give an 80% power to detect a very small correlation coefficient of 0.2 at a level of 0.05 [46]. For exploratory factor analysis (EFA), a sample size of 150 is sufficient if the dataset has several high factor loading scores (> 0.80) [40]. Validity assessments were done with a representative random sample of 200 participants living with chronic LBP in rural communities of Enugu State–as part of a larger population-based cross-sectional study of a representative sample of 200 participants living with chronic LBP in rural communities in Enugu State, South-eastern Nigeria.

As described in detail elsewhere [36, 46], multistage cluster sampling was used to select 10 rural communities (Oduma Ameke, Amagunze, Umuagama, Agbada Inyi, Edem Ani, Amagu-Uwenu, Mgbuji Eha-Amufu, Iheakpu Obollo Afor, Adaba Nkume, and Ukwa), representative of rural populations in Enugu State. The seventeen Local Government Areas (LGAs) in Enugu State were split into urban and rural LGAs. Enugu South, Enugu North and Enugu East are exclusively urban LGAs, and were excluded from the sampling frame. Of the remaining fourteen LGAs, ten LGAs were randomly selected with computer generated random numbers. This was to enable ten recruited research assistants, who were community health workers (CHWs), to collect data from 20 participants from each LGA. Each CHW was conveniently (familiarity with area) assigned to one of the selected ten LGAs. Each CHW randomly selected one community from each LGA by simple balloting, supervised by the lead author.

Village announcements were facilitated by the traditional head in each community. All eligible participants were stratified into males and females. Random selection by balloting (without replacement) was aimed at ensuring an equal representation of male and female participants. Overall, a sub-sample of twenty participants was selected in each of the ten communities, by asking participants to pick a folded paper from a pool of papers containing twenty yeses’ and the rest no’s. This resulted in a total of 200 participants.

#### Procedure

**Training community health workers for interviewer-administration of measures:** CHWs were required for data collection through interviewer-administration as a significant proportion of rural dwellers in Nigeria are not literate. They were recruited from the University of Nigeria Teaching Hospital (UNTH), Enugu.

A manual, based on the World Health Organisation Disability Assessment Schedule 2.0 guidelines for interviewer-administration of self-report measures [52], instructions by the developers of the measure, literature review, and findings from the verbal pretesting of the measure, was used for training. The CHWs were trained for two weeks, for interviewer-administration of the all the measures.

The training was daily, face-to-face, group-based, and done by the lead author. Measurement error was reduced by tailoring CHWs’ training to avoid asking questions in ways that could bias participants’ responses. Examples include avoiding the use of comments like ‘I know this might not apply to you…’). Training CHWs to assess all recruited participants whilst ensuring that no items or scales were unanswered prevented non-response errors.

**Data collection:** CHWs met with potential participants, provided information about the study and screened participants, by asking simple questions to rule out the ‘red flags’ for LBP. This excluded any LBP associated with underlying serious pathology, radiculopathy or spinal stenosis. This is in line with evidence-based guidelines for diagnosing LBP [53]. Informed consent was subsequently obtained. Participants were requested to describe their pain location with a body chart to confirm that pain was in the lower back. The Igbo-FABQ, BS-11 and the Igbo-RMDQ were then interviewer-administered. The Likert scales of the Igbo-FABQ and the eleven-point box scale (BS-11) [39, 40] were presented to participants as ‘flash cards’ as each item was read out.

To assess test-retest reliability, the Igbo-FABQ was completed at baseline on 11 August, 2014 among the convenient sample of 50 urban and rural Nigerian dwellers. Measurements were repeated seven days after first measurement. The same CHW collected data from each participant on the two occasions.

For validity assessment, the Igbo-FABQ, the BS-11 and the Igbo-RMDQ were completed at one time-point in a cross-sectional design on 22 August 2014 among the 200 rural dwellers.

The two samples were similar in characteristics except that the test–retest sample also included urban dwellers who routinely have higher literacy levels in Nigeria. However, recruiting different samples of rural and urban dwellers ensured wider applicability of the Igbo-RMDQ across rural and urban Nigeria, and all levels of literacy or illiteracy.

**Fidelity assessment:** Strategies were employed to avoid systematic differences in data collection by the community health workers. Only workers that passed the post-training examinations were recruited to facilitate adherence to data collection protocols. Furthermore, the lead author visited each CHW during data collection without prior arrangement and assessed their interviewing styles and data recording.

**Statistical analyses:** Statistical Package for Social Sciences version 22 (SPSS, Chicago, IL) was used. Data were assessed for normality using visual (normal distribution curve and Q-Q plot), and statistical methods (Kolmogorov-Smirnov, Shapiro-Wilk’s test and Skewness/Kurtosis scores). There was no need to handle missing data because the rigorous training of CHWs and interviewer-administration of measures ensured that no data were missing.

**Reliability:** Reliability assesses the ability of an instrument to measure consistently. Test–retest reliability evaluated how consistent the adapted FABQ consistently measured fear avoidance over time and was investigated using intra-class correlation coefficient (ICC).

ICC was calculated using a two-way random effects model (which assumes that measurement errors could arise from either raters or subjects), using an absolute agreement definition between test-retest scores. 0.7, 0.8 and 0.9 represented good, very good and excellent ICCs [54, 55]. Internal consistency (Cronbach’s alpha), which portrays the extent to which all items in a test measure the same construct, was calculated and rated as low/weak (0–0.2), moderate (0.3–0.6) and strong (0.7–1.0) [54].

Bland-Altman plots [56] were also used to visually assess the level of agreement between test-retest measurements by plotting mean scores against difference in total scores. Bland-Altman analysis accounted for the weakness of ICC which might indicate strong correlations between two measurements with minimal agreement.

Reliability was also evaluated using the standard error of measurement (SEM) and minimal detectable change (MDC). MDC is a statistical estimate of the smallest change detected by a measure that corresponds to a noticeable change in ability which is not due to measurement error. MDC was calculated using the standard error of measurement (SEM) which is based on the distribution method, and the reliability of the measure which takes precision into account [57]. SEM was based on the standard deviation (SD) of the sample and the test-retest reliability (R) of the Igbo-FABQ, and was calculated with Eq 1 below [57]:

$$SEM=SD\sqrt{\left(1-R\right)}$$ (1)

Eq 1: *Standard Error of Measurement*

MDC was subsequently calculated with Eq 2 below:

$$MDC=1.96\times \sqrt{2}\times SEM$$ (2)

Eq 2: *Minimal Detectable Change*

**Validity:** Construct validity evaluates the extent to which a measure assesses the construct it was intended to measure. As there are no “gold standard” Igbo fear avoidance measures, construct validity was investigated. Construct validity was assessed with Spearman’s correlation coefficients (data was not normally distributed), and was rated as weak (0–0.2), moderate (0.3–0.6), and strong (0.7–1.0) [58]. The BS-11 [39, 40], a one-item numeric pain intensity scale and the Igbo-RMDQ were used in the validity assessments informed by the established relationship between fear avoidance beliefs, pain intensity and self-reported disability in the literature. As fear avoidance beliefs assess pain-related fear [12, 17, 59, 60], Igbo-FABQ is expected to have at least a moderate correlation with pain intensity as suggested in the literature [59, 61–63]. Moreover, fear avoidance beliefs are predictors of self-reported disability in rural Nigeria [36]. There was no Igbo quality of life measure with which to validate the Igbo-FABQ.

Exploratory factor analyses (EFA) was the last psychometric analysis performed to determine the number of factors influencing the Igbo-FABQ, i.e. the items that go together (dimensionality) [64]. EFA was applied according to Kaiser Meyer Olkin (KMO) and the Bartlett’s test with a minimum eigenvalue for retention set at ⩾1.0 (Kaiser’s rule) [65]. Retained and excluded factors were also explored visually on a scree plot. Promax (oblique) rotation, which assumes that factors can be related, was done, and factor loadings less than 0.3 were suppressed as recommended [64]. Extraction was done using principal axis factoring. The number of factors and the underlying relationships between the items were then compared with the factor structures of the original FABQ to enhance an understanding of the differences in population (rural Nigerian versus western) characteristics.

**Floor and ceiling effects:** Ceiling or floor effect occurs when a high proportion of participants score the highest or the lowest score, respectively, implying that a measure is unable to discriminate between participants at either extreme of the scale. A ceiling or floor effect was defined as 15% or more of the total sample of 250 participants scoring 0 or 66 on the total score of the Igbo-FABQ [66].

## Results

### Cross-cultural adaptation

As the same sample were used in the cross-cultural adaptation of other Igbo self-report measures, the demographic characteristics of participants are the same as that reported elsewhere [46].

#### Participants

Slightly over half of the participants were males and manual workers. These included farmers, panel beaters and welders. Non-manual workers included civil servants and traders. Most participants were from the Pentecostal Christian religion, married, with secondary education. Half of them were literate in English only (Table 1).

**Table 1:** Demographic characteristics of participants that pre-tested the questionnaire.

n = 12	Frequency	%
Mean age = 45 years		
GENDER		
Male	7	58.33
Female	5	41.67
MAIN OCCUPATION		
Manual workers	7	58.33
Non-manual workers	5	41.67
RELIGION (CHRISTIAN DENOMINATION)		
Protestant Pentecostal	10	83.33
Catholic	2	16.67
MARITAL STATUS		
Married	11	91.67
Single	1	8.33
EDUCATIONAL LEVEL COMPLETED		
Secondary	4	33.33
Primary	3	25.00
None	3	25.00
Tertiary	2	16.67
LITERACY (ABILITY TO READ AND WRITE)		
Illiterate (inability to read and write)	4	33.33
English	6	50.00
English and Igbo	2	16.67

#### Translation, comprehensibility and cultural equivalence

The cross-cultural adaptation was straight forward. The expert review committee introduced a clause in the instruction: ‘or say the number’ to give the option of interviewer-administration. For interviewer-administration of the Igbo-FABQ, the Likert scales were shown to participants as flash cards and they were instructed to verbally select one option after the interviewer had read out each item. Item 8, ‘I have a claim for compensation for my pain’ was modified to the Igbo equivalent of ‘I have a compensation or gains I get from having my pain’ as there is no social benefit in rural Nigeria. The Igbo phrase for ‘physical activity’ could also mean ‘being active’ or ‘moving the body’ or ‘moving about’ and was used in the items with ‘physical activity’. During field verbal pretesting, participants were more likely to select anchors: 0, 3 and 6. The Igbo word for ‘waist pain’ was how participants understood LBP. Literal Igbo translation of LBP was understood as pain of the entire back. Therefore ‘waist pain’ was used in place of LBP. LBP was similarly understood as ‘waist pain’ in other rural African contexts. Participants did not find any item offensive.

### Psychometric properties

As the same sample were used in the psychometric testing of other Igbo self-report measures, the demographic characteristics of participants are the same as that reported elsewhere [46].

#### Fidelity results

As similarly reported elsewhere [46], the CHWs adhered to the interviewing styles underscored during the training. These included being neutral during interview, not responding by word or gesture, either positively or negatively to any responses; discouragement of digression, distraction and inappropriate queries and requests, and not changing the expression and sequence of questions or responses in the measures. Data recording was found to be adequate. For each item, the CHWs provided only one answer, and recorded in the space provided for each item in the measure.

#### Participants

The demographic characteristics of the two samples are presented in Tables 2 and 3. In Table 2, there is the test-retest sample of 50 participants. Most of the participants were females, married, in paid employment or self-employed. Slightly less than half were rural dwellers in Enugu state. Participants were mostly middle aged with secondary level of education. In Table 3, there are the 200 participants in the cross-sectional validity testing. The participants were all rural dwellers in Enugu state. Nearly equal numbers were males. They were middle aged with primary level of education. Most of them were married and self-employed.

**Table 2:** Demographic characteristics of participants that completed test-retest reliability testing.

n = 50	Frequency (%)	Mean (SD)
**Gender**		
Female	32 (64.0)	
Male	18 (36.0)	
**Habitation**		
Rural	20 (40.0)	
Urban	30 (60.0)	
**Age (years)**		45.2 (11.55)
**Education (years)**		13.3 (7.14)
**Current marital status**		
Currently married	37 (74.0)	
Never married	8 (16.0)	
Widowed	4 (8.0)	
Separated	1 (2.0)	
**Work status**		
Paid work	25 (50.0)	
Self-employed (own business or farming)	19 (38.0)	
Keeping house/homemaker	2 (4.0)	
Student	2 (4.0)	
Non-paid work (volunteer or charity)	1 (2.0)	
Unemployed (health reasons)	1 (2.0)	

**Table 3:** Demographic characteristics of participants that participated in the cross-sectional validity testing.

n = 200	n (%)	Mean (SD)
**Sex**		
Female	112 (56.0)	
Male	88 (44.0)	
**Age (years)**		48.6 (12.0)
**Education (years)**		7.0 (6.4)
**Current marital status**		
Currently married	143 (71.5)	
Widowed	31 (15.5)	
Never married	22 (11.0)	
Cohabiting	2 (1.0)	
Separated	2 (1.0)	
**Work status**		
Self-employed (own business or farming)	125 (62.5)	
Paid work	31 (15.5)	
Non-paid work (volunteer or charity)	16 (8.0)	
Keeping house/homemaker	13 (6.5)	
Student	7 (3.5)	
Unemployed (health reasons)	4 (2.0)	
Unemployed (other reasons)	3 (1.5)	
Retired	1 (0.5)	

#### Reliability

In Table 4, internal consistency was shown to be excellent (α = 0.86) for total scoring of Igbo-FABQ; good for physical activity (α = 0.81) and work (α = 0.80) subscales, and no item deletion increased internal consistency. Good intra class correlation coefficients were observed for total scoring (ICC = 0.72), physical activity (ICC = 0.71), and work (ICC = 0.72) subscales. Standard error of measurement and minimal detectable change were 7.40 and 20.51 for total scoring; 3.21 and 8.90 for the physical activity subscale; and 5.30 and 14.69 for the work subscale.

**Table 4:** Reliability of Igbo-FABQ.

**Igbo-FABQ total score** Number of items: 11; Cronbach’s alpha global score: 0.86; ICC (95% CI): 0.72 (0.51, 0.84)							
Cronbach’s alpha If Item Deleted							
**2**	**3**	**4**	**5**	**6**	**7**	**9**	**10**
0.86	0.85	0.84	0.84	0.84	0.84	0.86	0.84
**11**	**12**	**15**					
0.85	0.85	0.84					
SEM: 7.40 MDC: 20.51							
**Igbo-FABQ (physical activity)** Number of items: 4; Cronbach’s alpha global score: 0.81; ICC (95% CI): 0.71 (0.47, 0.84)							
Cronbach’s alpha If Item Deleted							
2	**3**	**4**	**5**				
**0.78**	0.74	0.78	0.75				
SEM: 3.21 MDC: 8.90							
**Igbo-FABQ (work)** Number of items: 7; Cronbach’s alpha global score: 0.80; ICC (95% CI): 0.72 (0.51, 0.84)							
Cronbach’s alpha If Item Deleted							
**6**	**7**	**9**	**10**	**11**	**12**	**15**	
0.75	0.76	0.77	0.76	0.77	0.78	0.80	
SEM: 5.30 MDC: 14.69							

**Fig 2:**
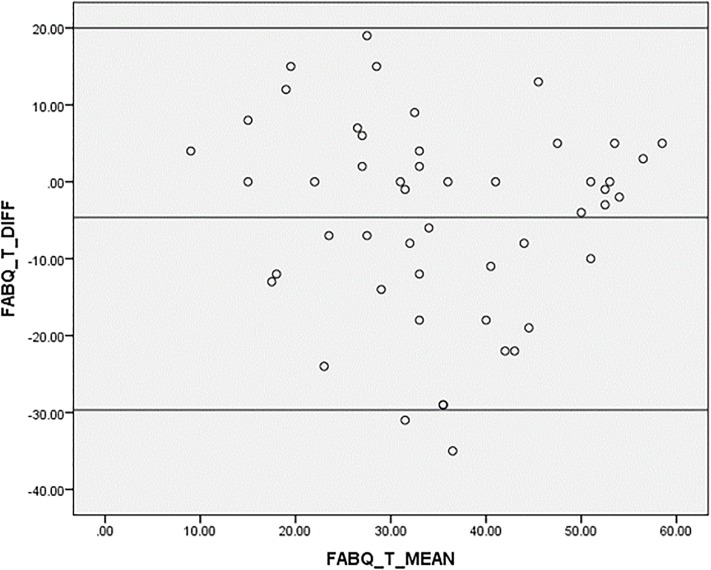
Bland-Altman plot for test-retest agreement of Igbo-FABQ (total). [upper limit: (+1.96 SD): 20.39; mean: -4.64 (-8.27, -1.01); SD: 12.77; lower limit: (-1.96 SD): -29.67].

**Fig 3:**
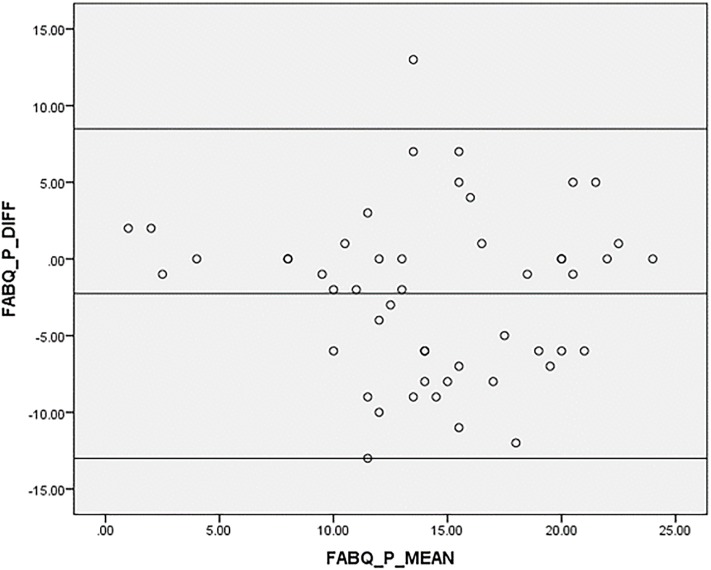
Bland-Altman plot for test-retest agreement of Igbo-FABQ (physical activity). [upper limit: (+1.96 SD): 8.48; mean: -2.26 (-3.81, -0.70); SD: 5.48; lower limit: (-1.96 SD): -13.00].

**Fig 4:**
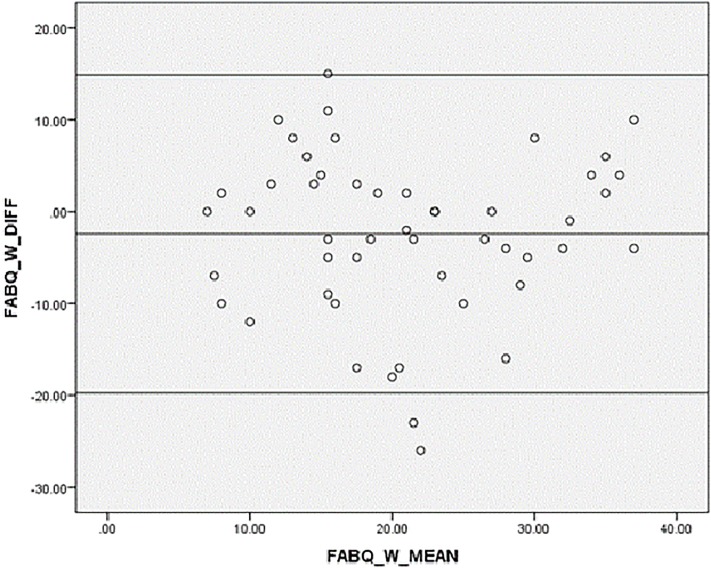
Bland-Altman plot for test-retest agreement of Igbo-FABQ (work). [upper limit: (+1.96 SD): 14.89; mean: -2.42 (-4.93, 0.09); SD: 8.83; lower limit: (-1.96 SD): -19.73].

In Figs 2–4, agreement was shown to be adequate between test-retest values of the Igbo-FABQ total score and its subscales as mean differences were close to zero, and most points were within 95% limits of agreement of the mean differences.

#### Construct validity

In Table 5, Igbo-FABQ and its subscales were illustrated to have moderate correlations with pain intensity (BS-11) and moderately high correlations with self-reported disability (Igbo-RMDQ).

**Table 5:** Spearman’s correlation between Igbo-FABQ, pain intensity and disability.

	Igbo-BS-11	Igbo-RMDQ
Igbo-FABQ (total)	0.36**	0.56**
Igbo-FABQ (physical activity)	0.28**	0.52**
Igbo-FABQ (work)	0.37**	0.53**

**p<0.01

#### Factor structure

A two-factor solution of the Igbo-FABQ was produced. 72.73% of the items had factor loadings above 0.5. 63.64% of the items loaded on their corresponding factor in the original measure: 71.43% for work subscale; 50.00% for physical activity subscale. Factor 1 had all but two items (12, 15) of the original work subscale loading on it, with additional two items of the original physical activity subscale (2, 3) loading on it. Factor 2 had two of the four items (4, 5) of the original physical activity subscale, and two items (12, 15) of the original work subscale loading on it (Table 6).

**Table 6:** Exploratory factor analysis of the Igbo-FABQ.

	1	2
FABQ9	.903	
FABQ6	.759	
FABQ11	.727	
FABQ7	.709	
FABQ10	.687	
FABQ2	.452	.404
FABQ3	.421	.354
FABQ5		1.004
FABQ4		.876
FABQ12		.562
FABQ15		.459
KMO = 0.91		
Χ^2^ = 1338.99***		

Only factor loadings above 0.3 are shown; KMO = Kaiser-Meyer-Olkin measure of sampling adequacy; χ^2^ = Bartlett’s test of sphericity tested with chi-square ***p<0.001; Extraction Method: Principal Axis Factoring; Rotation Method: Promax with Kaiser Normalization; Rotation converged in 3 iterations.

#### Ceiling and floor effects

None of the participants scored 0 and 66 on the total score of the Igbo-FABQ. 2% (4/200) and 3.5% (7/200) scored 0 and 24 on the FABQ-PA respectively. None of the participants and 0.5% (1/200) scored 0 and 42 on the FABQ-W respectively.

## Discussion

The cross-cultural adaptation, comprehensibility and acceptability of the Igbo-FABQ was very good, similar to other translations [67–71]. Item 8, ‘I have a claim for compensation for my pain’ which was skewed in a German population because most participants ‘completely did not agree’ with it [72], reflected the findings in this population as Nigeria lacked social benefits. The item was adapted to capture this reality. The Igbo phrase for ‘physical activity’ could also mean ‘being active’, ‘moving the body’ or ‘moving about’, all of which are in line with the fear avoidance model [12–15].

A range of Cronbach’s alpha between 0.80 and 0.86 of Igbo-FABQ and its subscales are in line with both the original measure [17], and other translations [37, 68, 71, 72].

Good reliability observed for Igbo-FABQ with ICCs ranging from 0.71 to 0.72, and Bland-Altman plots that suggested good agreement, are in line with the literature [17, 37, 68, 71, 72].

SEM of 3.21, MDC of 8.90, and limits of agreement of between -13.00 and 8.48 of the physical activity subscale of the Igbo-FABQ are all within the reported MCID of 13 of the physical activity subscale of the original measure [38]. This suggests good clinical utility of the Igbo-FABQ. However, MCID combines both anchor-based methods (patients’ rating of improvement) and distribution-based method (based on the SEM), and has not been determined in this population. MDC should be sufficiently small to detect MCID [57]. However MDC solely determined using distribution-based methods may lead to patients with actual improvement being rated as not improved [73], as measurement error is not constant across scores and populations [74].

The moderate correlations between Igbo-FABQ, its subscales, and pain intensity and self-reported disability support the literature [12, 14, 47, 59, 61–63, 71] and suggest construct validity of the measure. The lack of any Igbo quality of life measure with which to validate the Igbo-FABQ is a limitation. However, the use of the Igbo-RMDQ may mitigate this limitation as individuals’ perception of their functional ability may reflect how chronic back pain impacts on quality of daily life [47]. A two-factor structure of the Igbo-FABQ was produced similar to the original measure [17] and Norwegian adaptation [68]. However, the physical activity factor was not precise as half of the items also loaded on the work subscale, in contrast to findings in a German population [72]. This may be due to the fact that most rural dwellers (from whom the factor structures were determined) were manual workers. It is therefore possible that they could not distinguish between physical activity and work as their job activities involved physical movements and activity. This lack of distinction between work-related activities and physical activity was also suggested in a previous qualitative study in this population [35]. Therefore, total scoring, rather than the subscales of the Igbo-FABQ may be more useful in such populations of manual labourers in rural Nigeria.

The strength of this study is that it enabled the development of a valid and reliable measure of fear avoidance beliefs for Igbo speaking populations that included illiterate people often neglected despite being the most vulnerable group with the worst health outcomes. The demonstrated complexity of developing valid and reliable measures for this population could be related to cultural, linguistic and literacy issues.

Despite acceptable validity and reliability levels, high sample variability and measurement errors may have been introduced by low literacy rates, interviewer-administration in place of self-administration, and data collection by several raters. This is important considering that MDC not only depends on the inherent measurement error of an instrument, but varies across populations and contexts [73, 75]. In view of this, sensitivity-to-change studies of the Igbo-FABQ are required in populations of varying literacy levels, with single raters, and including more rigorous analysis such as receiver operating characteristic (ROC) curves, which includes patients’ own global impression of change. These studies need to confirm the MDCs, and determine the proportion of people that achieve the MDCs of the Igbo-FABQ. Future studies should include bilingual testing involving both the original FABQ and the Igbo-FABQ, which incorporates item by item agreement, in populations with adequate literacy levels to enable comprehension of English and Igbo. Furthermore, confirmatory factor analysis of the Igbo-FABQ, which would require a sample size of at least 300 when there are only a few high factor loading scores (> 0.80) [64], should be done in future research. The small number of Igbo measures with which to validate the Igbo-FABQ is a potential weakness. However, validity of the Igbo-FABQ is supported by correlations that are in line with established literature. The Igbo-FABQ can therefore be used to validate other fear avoidance beliefs measures and quality of life measures in similar populations.

## Conclusions

The Igbo-FABQ (S1 Table) is valid and reliable for clinical and research purposes in Igbo speaking culture. It would support global health initiatives which often involve concurrent activities in countries of different languages and culture.

## Supporting information

S1 Table The Igbo Fear Avoidance Beliefs Questionnaire (Igbo-FABQ). (PDF)
