# Supplementary material for: Translation, cultural adaptation and psychometric testing of Igbo fear avoidance beliefs questionnaire in mixed rural and urban Nigerian populations with chronic low back pain
Source: PLoS One. 2019 May 14;14(5):e0216482. doi: 10.1371/journal.pone.0216482 (PMC6516646; doi:10.1371/journal.pone.0216482)
Supplement: S1 Table — (PDF) [file pone.0216482.s001.pdf]

Igbo Fear Avoidance Beliefs Questionnaire

***Chinonso N Igwesi-Chidobe, Charity Amarachukwu, Isaac O Sorinola and Emma L Godfrey**

Nke a bu ufodu ihe ndi oria ndi ozo gwara anyi maka ahu mgbu ha. Kanye ihe ma o bu kwuo onu ogu nke o bu site na efu ruo na isii (0-6) iji kowaa udi mmeghari ahu dika ihulata ala, ibuli ihe, iga ije ma o bu inya ugbo ala ga esi emetuta ukwu mgbu gi.

		Ekweghi kpamkpam			Amach -aghi			Ikwere kpamkpam
1.	Ihe mgbu m bu Mmeghari ahu butere ya	0	1	2	3	4	5	6
2.	Mmeghari ahu na eme ka mgbu m kara njo	0	1	2	3	4	5	6
3.	Mmeghari ahu nwere ike inye m mmeru ahu na ukwu m.	0	1	2	3	4	5	6
4.	Ekwesighi m ime mmeghari ahu maka na o nwere ike ime ka mgbu m kara njo	0	1	2	3	4	5	6
5.	Enweghi m ike ime mmeghari ahu maka na o nwere ike ime/na eme ka mgbu m kara njo.	0	1	2	3	4	5	6

Ihe ndi a edeputara gbasara etu oru I na aru si emetuta ukwu mgbu gi ma o bu etu o ga esi metuta ukwu mgbu gi

		Ekweghi kpamkpam			Amach -aghi			Ikwere kpamkpam
6.	Mgbu m bu oru m na aru butere ya ma o bu ihe mberede na ulo oru.	0	1	2	3	4	5	6
7.	Oru m na aru na eme ka mgbu m ka njo.	0	1	2	3	4	5	6
8.	E nwere m ihe i ti aka na obi/ ma o bu uru m na enweta maka mgbu m.	0	1	2	3	4	5	6
9.	Oru m na aru bu oru ike nke ukwuu.	0	1	2	3	4	5	6
10.	Oru m na aru na eme ma o bu ga eme ka mgbu m ka njo.	0	1	2	3	4	5	6
11.	Oru m na aru nwere ike i meru m ahu na ukwu.	0	1	2	3	4	5	6
12.	Ekwesighi m i bu ihe mgbu nke na egbu m ugbu a na aru oru m na aru.	0	1	2	3	4	5	6

13. Enweghi m ike ibu mgbu a m na enwe ugbu a na aru oru m na aru.	0	1	2	3	4	5	6
14. Enweghi m ike iru oru m na aru ruo mgbe ihe mgbu m natara ogwugwo.	0	1	2	3	4	5	6
15. Echeghi m na m ga alaghachi na oru m na aru n'ime onwa ato.	0	1	2	3	4	5	6
16. Echeghi m na m ga enwe ike ilaghachi ozo na oru m na aru.	0	1	2	3	4	5	6
